# Polymorphisms −374 T/A and −429 T/C of the Receptor for Advanced Glycation End-Products (RAGE) and Serum Levels of RAGE (sRAGE) Are Not Associated with Metabolic Syndrome

**DOI:** 10.3390/metabo13040521

**Published:** 2023-04-05

**Authors:** Diana Elizabeth González-Guerrero, Maria-Luisa Lazo-de-la-Vega-Monroy, Armando Gómez-Ojeda, Claudia Luévano-Contreras, Armando Rojas-Rubio, Ma. Eugenia Garay-Sevilla

**Affiliations:** 1 Department of Medical Science, Division of Health Science, University of Guanajuato, Campus León, León 36000, Mexico; 2 Biomedical Research Laboratory, Medicine Faculty, Catholic University of Maule, Talca 3605, Chile

**Keywords:** RAGE, sRAGE, metabolic syndrome, polymorphisms −374 T/A, −429 T/C

## Abstract

RAGE is a multi-ligand transmembrane glycoprotein that promotes biological signals associated with inflammatory responses and degenerative diseases. sRAGE is a soluble variant that has been proposed as an inhibitor of RAGE activity. The −374 T/A and −429 T/C polymorphisms of the advanced glycation end-product receptor *AGER* gene have been associated with the development of some diseases, such as types of cancer, cardiovascular disease, and micro- and macro-vascular disease in diabetes, among others, but their role in metabolic syndrome (MS) is still unknown. We studied 80 healthy males without MS, and 80 males with MS, according to the harmonized criteria. The −374 T/A and −429 T/C polymorphisms were genotyped by RT-PCR, and sRAGE was measured by ELISA. Allelic and genotypic frequencies did not differ between the non-MS and MS groups (−374 T/A *p* = 0.48, *p* = 0.57 and −429 T/C *p* = 0.36, *p* = 0.59, respectively). Significant differences were found in fasting glucose levels and diastolic blood pressure in the genotypes of the −374 T/A polymorphism in the non-MS group (*p* < 0.01 and *p* = 0.008). Glucose levels were different in the −429 T/C genotypes in the MS group (*p* = 0.02). The sRAGE levels were similar in both groups, but the non-MS group showed a significant difference between individuals with only 1 or 2 components of metabolic syndrome (*p* = 0.047). However, no associations of any SNP with MS were found (recessive model *p* = 0.48, dominant model *p* = 0.82 for −374 T/A; recessive model *p* = 0.48, dominant model *p* = 0.42 for −429 T/C). The −374 T/A and −429 T/C polymorphisms were not associated with MS in a Mexican population and had no influence on serum sRAGE levels.

## 1. Introduction

Metabolic syndrome (MS) is defined as a group of biochemical, physiological, and anthropometric abnormalities, such as hyperglycemia, dyslipidemia, elevated blood pressure, and abdominal obesity, that can considerably increase the risk of an individual to develop cardiovascular disease (CVD) and type-2 diabetes mellitus (T2DM) [1].

Advanced glycation end-products (AGEs) are among the possible risk factors for developing MS. AGEs are a very wide, complex, and heterogeneous group of molecules with pro-oxidant and pro-inflammatory properties. When accumulated in the body, AGEs can alter the structure and the function of proteins by modifying their biological activities, particularly by crosslinking them with intracellular and extracellular matrix proteins. Furthermore, the accumulation of AGEs promotes the activation of intracellular signals through mechanisms mediated by receptors [2]. Of these, the receptor for advanced glycation end-products (RAGE) is the most widely studied. 

RAGE is a multi-ligand transmembrane cellular surface protein that interacts with several molecules in addition to AGEs, such as Mac-1, S100/calgranulines, HMGB1, oxLDL, DNA, and RNA, among others. These interactions trigger the secretion of several kinases that, subsequently, activate NF-kB, stimulating inflammation and tissue damage [3]. The sustained activation of the AGE–RAGE axis favors the development of inflammatory, neurodegenerative, and autoimmune diseases, as well as cancer, CVD, T2DM, and their complications [4].

RAGE also presents as other isoforms. One of them, known as sRAGE, originates by alternative splicing or by the proteolytic cleavage of RAGE, liberating an extracellular domain of the full-length RAGE [5,6]. The role of sRAGE is not completely understood. Investigations in humans and animal models have reported that a decrease in the circulating sRAGE was associated with a variety of pathologies, such as coronary arterial disease (CAD) [7], neurodegenerative disorders [8,9,10], autoimmune diseases [11,12], cancer [13,14], non-alcoholic fatty liver disease [15], and morbid obesity [16], among others. On the other hand, elevated circulating sRAGE levels have also been associated with a decrease in the estimated glomerular filtration rate [17] and other inflammatory molecules, such as HMGB1 and AGEs [18]. In metabolic syndrome, sRAGE concentration decreased as the components of the syndrome increased [19,20,21,22]. However, other studies have reported an elevation of sRAGE in metabolic syndrome [23].

It has been proposed that the serum levels of sRAGE could be modified by many factors, such as the polymorphisms in *AGER*, the gene that encodes the RAGE protein [24]. This gene is highly polymorphic and contains 11 exons and 10 introns, and it is in chromosome 6p21.3 [25]. Two of the most studied polymorphisms in *AGER*, −374 T/A (rs1800624) and −429 T/C (rs1800625), are found in the promoter region, and they have been shown to influence its transcriptional activities [26]. The A-allele of the −374 T/A polymorphism has been suggested as a protective factor of CVD [27,28,29], as well as for diabetes mellitus and its complications [30,31]. On the other hand, the −429 T/C polymorphism has been associated with diabetic retinopathy [26], insulin resistance [32], coronary artery disease [33], and diabetic nephropathy [34]. Nevertheless, other studies have reported contradictory results regarding both polymorphisms [30,35,36,37,38,39,40,41,42]. 

It has been suggested that other SNPs in *AGER* may influence sRAGE levels, possibly regulating alternative splicing sites and modulating RAGE’s susceptibility to proteolytic cleavage [43]. To date, −374 T/A and −429 T/C have not been studied in relation to metabolic syndrome, neither have they been studied in Mexican population. Hence, the aim of the present study was to evaluate the association of these polymorphisms and serum concentrations of sRAGE, with the presence of metabolic syndrome in a Mexican population.

## 2. Materials and Methods

A cross-sectional study, approved by the ethics committee of the Universidad de Guanajuato (CIBIUG-P41-2017) was performed. Written informed consent was obtained from all participants before enrollment.

### 2.1. Participants

The sample size was calculated according to the Armitage trend test, taking into account the prevalence of disease (0.5), the frequency of the risk alleles of the polymorphisms (rs1800624 = 0.258, rs1800625 = 0.094), a type-I error of 0.05, a power of 80%, a case-control ratio of 0.5, and an additive model. According to the calculations obtained, we doubled the sample size, hoping for a greater statistical power, and then we studied 160 non-related males from the central region of Mexico [44], who met the following criteria: aged 25–45 years old; absence of T2DM, CVD, chronic or acute infection, or renal disease; an intake of <3 cigarettes/day [45]; and <30 g alcohol/day [46]. Volunteers were divided into 2 groups: with (MS, *n* = 80) and without MS (non-MS, *n* = 80), according to the harmonized criteria [1,47]. Participants with at least 3 of the following 5 conditions were included in the MS group: (I) Waist circumference ≥ 94 cm; (II) Systolic blood pressure > 130 mmHg and/or diastolic blood pressure > 85 mmHg or use of anti-BP medication; (III) High-density lipoprotein (HDL) cholesterol < 40 mg/dL; (IV) Triglycerides ≥ 150 mg/dL or use of medications for high triglycerides; and (V) Fasting blood glucose ≥ 100 mg/dL or use of medications for high glucose. Volunteers without any MS criteria were included in the non-MS group. 

### 2.2. Data Collection

Clinical history, physical examination, and anthropometric measurements were obtained for all subjects. Height and weight were measured using a SECA stadiometer and a SECA scale respectively, and body mass index (BMI) was calculated as kilogram per square meter (kg/m^2^). Waist circumference (WC) was measured using Lufkin^®^ metallic tape, (Crescent Lufkin, Saginaw, Michigan, USA) and blood pressure was measured with an Omron HEM-7320-LA electronic monitor (Omron Healthcare Co. Ltd., Kyoto, Japan), twice in the right arm after a 5 min rest in a sitting position, and the average of the 2 measurements was recorded. All evaluations were performed by trained personnel.

### 2.3. Blood Sampling and Analyses 

Venous blood was collected from participants after overnight fasting to measure serum glucose concentrations using glucose GOD-POD (Lakeside, Mexico City), triglycerides, total cholesterol and HDL cholesterol were measured using enzymatic methods in an autoanalyzer (Spinreact-Spinlab, Model 6002390-412-02 Giron Spain). Creatinine was measured by inmunoturbidimetry (Spinreact, Spain). Serum aliquots were stored at −80 °C until further determination of sRAGE, which was measured by ELISA, according to the manufacturer’s instructions (R&D Systems). Urinalysis was performed on each sample using a colorimetric method with a urine test strip (Roche).

### 2.4. Genomic DNA Isolation 

Total DNA was extracted from whole blood samples using phenol-chloroform. DNA concentration and quality were assessed by spectrophotometry (Nanodrop 2000, ThermoScientific, Waltham, MA, USA). Additionally, agarose gel electrophoresis was carried out to assess the integrity of extracted DNA. Each DNA sample was diluted to a final concentration of 5 ng/µL prior to the genotyping assays.

### 2.5. AGER Genotyping Assay 

Two genomic variants of the *AGER* gene were studied: −374 T/A (rs1800624) and −429 T/C (rs1800625). Genotyping for each subject was performed using Taq Man SNP genotyping assays (assay ID for rs1800624 C___3293837_1_, and C___8848033_1_ for rs1800625, Applied Biosystems, Foster City, CA, USA), and was carried out on a CFX96 PCR instrument (Bio-Rad, Hercules, California, USA) according to manufacturer’s protocol. Conditions of the assay and result analysis were performed with the Bio-Rad CFX Manager v3.1 software (Bio-Rad Laboratories, Hercules, CA, USA): 95 °C for 10 min, 50 cycles of 95 °C for 15 s, 60 °C for 1 min and 95 °C for 15 s, and finally 4 °C for 5 min. 

### 2.6. Statistical Analysis

Normality of data was evaluated with the Lilliefors Kolmogorov-Smirnov test. Since most of the variables did not present a normal distribution, data were reported as median and interquartile ranges. To evaluate differences between the MS and non-MS groups, the Mann-Whitney U test was used. 

Genotypic and allelic frequencies were determined, and the Hardy-Weinberg equilibrium of each variant was calculated using X^2^ with Haploview v4.2 software..

To evaluate clinical and metabolic variables, as well as sRAGE levels, according to genotype, the Kruskal-Wallis test with multiple comparisons was used. 

To test if the effect of the evaluated SNPs could be cumulative, we constructed an unweighted genetic risk score based on the number of risk alleles carried by each subject for each of the two SNPs, assuming that both SNPs could contribute equally. The A allele of the −347 T/A polymorphism, and the C allele of the −429 T/C polymorphism were considered as risk alleles. One point was added for each risk allele; thus, each subject could have a score from 0 (no risk alleles for any SNP) to 4 (both SNPs heterozygous for the risk allele). This genetic risk score was used as a continuous variable to test for the association with MS in non-MS and MS groups with the Mann–Whitney U test. Furthermore, serum sRAGE levels in each group of the genetic risk score were analyzed with the Kruskal-Wallis test.

To evaluate the association of each polymorphism with the MS under the dominant and recessive models, the X^2^ test with Yates correction was used.

The haplotype analysis was performed with X^2^ using Haploview v 4.2 software. All other tests for the statistical analysis were performed using Statistica v 7.0 software (Statsoft Inc., Tulsa, OK, USA). Significance was defined as a value of *p* < 0.05 for all analyses.

## 3. Results

Males with and without metabolic syndrome were studied, 80 in each group. The clinical and metabolic characteristics of both groups are shown in Table 1. The age of the participants was 34.5 ± 9.5 in the non-MS group, and 37 ± 10 in the MS group. As expected, the MS group had higher waist circumferences; blood pressure results; glucose and triglycerides levels; and lower HDL-cholesterol levels, than the non-MS group. There were no differences in the serum sRAGE levels between the non-MS and MS groups.

### 3.1. Descriptive Analysis of the SNPs 

The genotypic distributions of the polymorphisms were evaluated by Hardy-Weinberg equilibrium (*p* > 0.05). The two genetic variants were in linkage equilibrium (D’ = 1.0, *p* = 0.032). The genotypic and allelic distributions of the −374 T/A and the −429 T/C variants did not differ significantly between the non-MS and MS groups (−374 T/A *p* = 0.57, −429 T/C *p* = 0.59) (Table 2).

**Figure 1:**
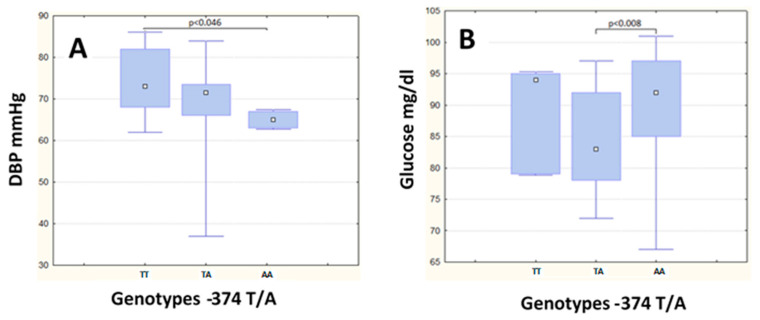
Diastolic blood pressure (DBP) and glucose levels in the −374 T/A SNP in non-MS group. Differences in (**A**) Diastolic Blood Pressure and (**B**) Glucose levels among genotypes of the −374 T/A SNP in the non-MS group.

In the non-MS group, the subjects carrying the AA genotype of the −374 T/A polymorphism had lower DBP, as compared to the TT genotype (*p* < 0.046) (Figure 1A). On the other hand, those with the AA genotype had higher glucose levels, as compared to the TA genotype (*p* = 0.008) (Figure 1B). Age, waist circumference, BMI, and SBP, as well as the serum levels of creatinine, triglycerides, cHDL, and sRAGE did not differ between the TT, TA, and AA genotypes of the −374 T/A polymorphism, in the non-MS group. No statistically significant differences between these genotypes were observed in the MS group (Table 3).

**Figure 2:**
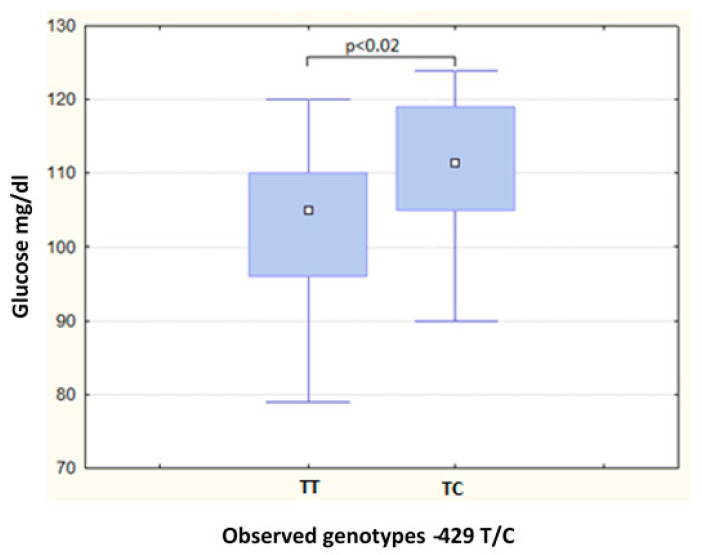
Differences in glucose levels among genotypes of −429 T/C SNP in subjects with MS.

In relation to the −429 T/C polymorphism, the TC genotype showed higher glucose levels in the MS group (*p* = 0.02) (Table 4, Figure 2). In contrast, any significant differences by genotype were found in the non-MS group.

The serum levels of sRAGE were similar between the non-MS and MS groups. In addition, no differences were observed when comparing sRAGE levels by genotypes between the non-MS and MS groups, for either of the two polymorphisms (Table 3 and Table 4).

### 3.2. Association of the Polymorphisms with MS 

When performing an association analysis based on the assumptions of the different inheritance models, none of SNPs studied were associated with MS under recessive- or dominant-inheritance genetic models (*p* = 0.48 for recessive model and *p* = 0.82 in dominant model for the −374 T/A SNP, and *p* = 0.48 and *p* = 0.42, respectively, in the −429 T/C polymorphism).

### 3.3. Genetic Risk Score and Haplotypes

In order to test if the combined SNPs could have a potential effect on any of the metabolic variables, we assigned an unweighted genetic risk score to each subject. Only scores of 0, 1, or 2 were presented due to the small number of risk-related allele heterozygotes. No associations among the genetic risk scores with the presence of MS were found. (*p* = 0.82). 

Similarly, when the haplotypes of both SNPs were generated using Haploview software, no differences in their frequencies were found between the MS and non-MS subjects (Table 5).

**Figure 3:**
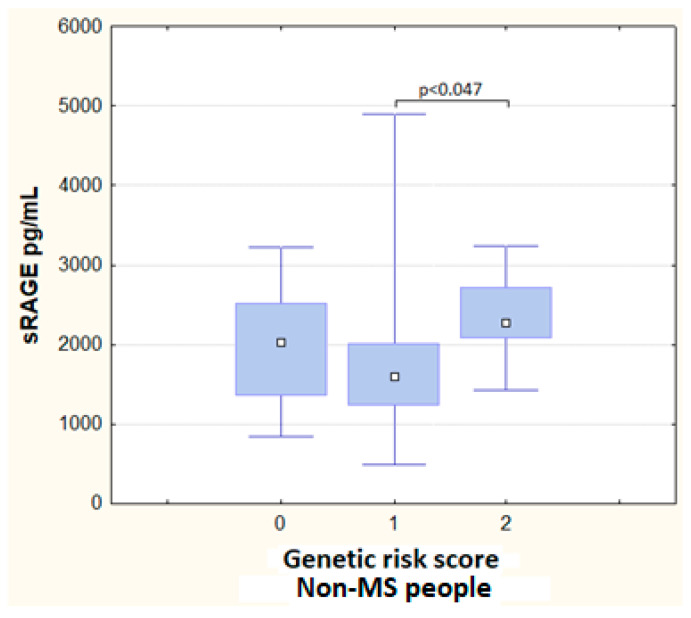
Serum levels of sRAGE by genetic risk score in non-MS subjects.

However, when comparing the circulating sRAGE levels between the genetic risk scores, a significant difference was observed in the non-MS group, as the sRAGE levels increased in subjects with two risk alleles (score 2) (*p* = 0.047) (Figure 3). No differences in the sRAGE levels by genetic risk scores were found in the non-MS group (*p* = 0.54).

## 4. Discussion

Polymorphisms in the *AGER* gene, −374 T/A (rs1800624) and −429 T/C (rs1800625), have been associated with several metabolic conditions, but their role in metabolic syndrome (MS) has not been established. 

An association of the A-allele of the −374 T/A polymorphism with lower levels of diastolic blood pressure had been previously reported in patients with diabetes, suggesting this allele could be a protective factor of CVD [27,29,48,49,50]. Our results were in line with this evidence, showing lower levels of diastolic blood pressure for the subjects with the AA genotype, albeit in the group without MS. The subjects with metabolic syndrome did not show significant differences in diastolic blood pressure levels. Similarly, Engelen et al. [51] reported that in females and males with normal glucose metabolism, the AA genotype of −374 T/A polymorphism was associated with lower levels of DBP, along with lower levels in other parameters of CVD. Interestingly, Engelen et al. also observed that this effect was inverted in people with alterations in their glucose metabolism, which agreed with our observation of higher glucose levels in the AA subjects without MS.

In the present study, the TA genotype of the −374 T/A polymorphism showed lower glucose levels in the non-MS group. It had been reported previously in Caucasian females that the A-allele of the polymorphism was associated with lower glucose levels [52]. It could be suggested that, in our study, the A-allele could be associated with lower levels of glucose. Nevertheless, this was not confirmed with the AA genotype, probably because it was barely found, in comparison with the other two genotypes. Regarding the −429 T/C polymorphism, no associations in the metabolic or clinical characteristics and the genotype were found in the non-MS group. However, TC-genotype carriers presented higher glucose levels. Wang et al. reported that the −429 T/C polymorphism was associated with an impaired glucose metabolism risk in primary hypertensive Chinese patients [53]. In contrast, a study of a subject with obesity and MS found no differences in the glucose levels associated with the genotypes of −374 T/A and −429 T/C polymorphisms [54].

In our study, no associations were found with these polymorphisms, alone or in haplotypes, and the presence of MS. Mehta et al. also reported that a haplotype with the A-allele of the −374 T/A polymorphism was associated with a higher risk of NASH but not with MS [54].

The −374 T/A polymorphism has been associated with changes in glucose metabolism, even at early stages of life [52]. Under normoglycemic conditions, the A-allele of the −374 T/A polymorphism increased the *AGER* transcription in vitro [26], while in hyperglycemia, the T-allele of the −374 T/A polymorphism appeared to behave similarly [55]. Therefore, our study could suggest that the A-allele of the −374 T/A polymorphism was not associated with an increase in the *AGER* transcription in normoglycemic people but, instead, rather associated with higher glucose levels and lower diastolic blood pressure values. Together with this evidence, our results highlighted the importance of the metabolic milieu in the influence of the *AGER* polymorphisms, particularly in glucose metabolism.

This study was performed exclusively in males. In this research, sex as a possible confounding variable was relevant because estrogens affect several metabolic processes and functions related to the components of MS [56,57,58]. Moreover, its role in relation to the AGE-RAGE axis has been controversial, as they have been associated with vascular dysfunctions [59], but also with a potential anti-inflammatory and protective role [60]. On the other hand, the association of lower testosterone levels in males with a greater number of MS components was also reported [61,62]. Therefore, sex could affect both the AGE–RAGE axis and the disease. A study suggested that a differential regulation existed in males and females in the process of chronic inflammation developed during MS [63].

The allele frequencies observed in our study were similar to those previously reported that studied these polymorphisms in T2DM and related complications in other populations [30,31,48,64,65]. However, in genetic association studies, it has been common that the association of a certain polymorphism with the disease of interest in different populations has often not been replicable, and this could be explained by the different frequencies of the polymorphisms, depending on the genetic background of the ethnic group of the study population. More than 90% of the current Mexican population is the result of a complex process of mixing between the native Indigenous people of the Americas and Europeans, substantially Spanish, which has resulted in the mestizo population [66]. Although it is documented that there has been variation in the frequencies of alleles associated with a disease among disparate populations, such as Mexico, this variation has been quite small [67]. However, a recent study reported an ethnicity-dependent contribution of *AGER* gene in the pathogenesis of diseases, such as type-2 diabetes [68]. 

The −429 T/C polymorphism could have a regulatory role in fasting blood glucose, sRAGE, and the development of impaired glucose metabolism, in primary hypertensive patients [53]. However, in our study, the sRAGE levels were only higher when comparing them between the genetic risk scores in the non-MS subjects. More research is needed to understand the specific mechanisms that drive the generation of sRAGE. Because of the pro-inflammatory state in MS, the expression of the RAGE protein increases and, consequently, the concentrations of sRAGE, as well. However, no differences were found in sRAGE levels when comparing the two groups. There was a possibility that the effect size of the unfavorable alleles had been overestimated, since although the SNPs included in the risk scores were in linkage equilibrium, the correlations between polymorphisms could have redounded the association, reducing the predictive performance of the model [69]. Therefore, future research could consider using only one SNP as a marker in an area of high LD and to include new loci identified in other regions of the *AGER* gene.

Moreover, significant differences in sRAGE serum levels have been reported when comparing multiethnic populations [70,71] although the underlying mechanism behind these differences had not yet been determined. In this regard, it was important to highlight that the soluble RAGE was derived from either the proteolytic cleavage (sRAGE) or the alternative splicing (esRAGE), and both forms were believed to function as decoy receptors. Although the ELISA kit used to quantify the soluble RAGE in this study was able to detect both forms, esRAGE represented only about 20% of the soluble RAGE receptors [72].

Additionally, the metabolic environment could have affected both the proteolytic cleavage mechanisms and the alternative splicing, and these same mechanisms could behave differently in different types of cells [73]. Moreover, the possibility that sRAGE and esRAGE fulfill different functions in the body should be considered [7]. Furthermore, measuring the enzymatic activity of the proteins responsible for the proteolytic cleavage of the RAGE receptor could help to clarify the production and function of sRAGE [7].

Furthermore, it was proposed that serum levels of sRAGE could have been modified by many factors, such as by the polymorphisms in *AGER* regulating alternative splicing sites or modulating RAGE’s susceptibility to proteolytic cleavage [43]. Additionally, in vitro, these polymorphisms were associated with an increase in *AGER*’s transcriptional activity.

Our study had some limitations. First, the number of polymorphisms studied in the population was reduced, and it was very likely that the −374 T/A and −429 T/C SNPs were in linkage disequilibrium with others in the same gene, or even with other genes in the major histocompatibility complex III (MHC), for they shared the same loci [36]. Second, the RAGE ligands related to metabolic stress were not measured, and this data could have provided a clearer vision of sRAGE in MS. Furthermore, it was proposed that simultaneous measurements of AGE and sRAGE as a ratio should be considered as a better biomarker/risk marker [74]. Third, neither the activities of enzymes, such as MMPs and ADAM10, involved in the proteolytic cleavage of RAGE, nor the RAGE mRNA transcription were measured. These could clarify the outlook on the production and the function of sRAGE. Fourth, the sample size did not allow us to have the necessary statistical power to observe the differences between the groups in the two polymorphisms studied, and the changes in the glucose levels were small in the non-MS group, according to the genotypes of the −374 T/A polymorphism, and therefore, the clinical significance was limited.

## 5. Conclusions

Our study showed that the −374 T/A and −429 T/C SNPs in *AGER* were not associated with MS, at least not in MS subjects in a Mexican population, and had no influence on the serum sRAGE levels. Therefore, future studies should be conducted with larger sample sizes regarding other polymorphisms that may be related to the different components of MS and could stratify the differences between males and females.

## Figures and Tables

**Table 1:** Clinical and metabolic characteristics of the study groups.

Variables	Non-MS Group *n* = 80 Median (IQR)	Metabolic Syndrome Group *n* = 80 Median (IQR)	*p*
Age (years)	34.50 (9.5)	37.00 (10.0)	0.008
BMI (kg/m^2^)	23.87 (2.9)	29.80 (5.0)	<0.001
Waist circumference (cm)	84.90 (7.2)	100.75 (10.3)	<0.001
SBP (mmHg)	109.00 (15.5)	126.00 (18.0)	<0.001
DBP (mmHg)	72.00 (10.0)	79.50 (12.0)	<0.001
Creatinine (mg/dL)	1.00 (0.2)	1.10 (0.2)	0.249
Glucose (mg/dL)	90.00 (16.5)	106.00 (13.5)	<0.001
Triglycerides (mg/dL)	85.50 (48.5)	208.00 (113.0)	<0.001
HDL-C (mg/dL)	47.00 (10.0)	33.00 (7.0)	<0.001
sRAGE (pg/mL)	1883.90 (955.5)	1989.21 (1156.4)	0.137

All values are expressed as median (M) and interquartile ranges. (IQR). BMI = Body mass index. SBP = Systolic blood pressure. DBP = Diastolic blood pressure. HDL-C = High-density lipoprotein cholesterol.

**Table 2:** Allelic and genotypic distributions of SNPs in the *AGER* gene between MS and non-MS groups.

SNP	Alleles	Non-MS Group		MS Group		*p*	Genotypes	Non-MS Group		MS Group		*p*
		*n*	%	*n*	%			*n*	%	*n*	%	
−374 T/A	T	122	76	115	73	0.48	TT	45	56	42	53	0.57
	A	38	24	43	27		TA	32	40	31	39	
							AA	3	3.8	6	7.6	
−429 T/C	T	141	88	146	91	0.36	TT	62	78	67	84	0.59
	C	19	12	14	8.8		TC	17	21	12	15	
							CC	1	1.3	1	1.3	

**Table 3:** Clinical and metabolic variables according to genotypes of the −374 T/A polymorphism.

	Non-MS Group							MS Group						
	TT		TA		AA		*p*	TT		TA		AA		*p*
	*n* = 45		*n* = 32		*n* = 3			*n* = 42		*n* = 31		*n* = 6		
	Median	(IQR)	Median	(IQR)	Median	(IQR)		Median	(IQR)	Median	(IQR)	Median	(IQR)	
Age (years)	33.00	(9.00)	36.50	(10.50)	33.00	(16.0)	0.66	37.00	(10.00)	39.00	(9.00)	36.00	(5.00)	0.74
WC (cm)	84.50	(9.40)	85.10	(6.00)	87.50	(17.1)	0.64	100.50	(12.30)	101.10	(11.50)	98.75	(5.50)	0.67
BMI (kg/m^2^)	23.86	(3.50)	23.87	(2.80)	25.22	(5.2)	0.56	29.27	(5.20)	30.10	(5.60)	29.32	(2.30)	0.84
SBP (mg/dL)	110.00	(19.00)	107.50	(13.00)	99.67	(27.0)	0.22	125.00	(19.00)	132.00	(20.00)	124.50	(4.00)	0.52
DBP (mg/dL)	73.00	(14.00)	71.50	(7.50)	65.00	(4.0)	0.008 ^a^	79.00	(14.00)	84.00	(10.00)	75.00	(12.00)	0.24
Glucose (mg/dL)	92.00	(12.00)	83.00	(14.00)	94.00	(16.0)	0.01 ^b^	107.00	(14.00)	105.00	(10.00)	107.00	(17.00)	0.88
Creatinine (mg/dL)	1.00	(0.20)	1.05	(0.20)	1.00	(0.3)	0.43	1.05	(0.20)	1.10	(0.30)	1.15	(0.60)	0.57
Triglycerides (mg/dL)	93.00	(50.00)	82.50	(46.00)	64.00	(70.0)	0.34	203.00 ^c^	(88.00)	228.00	(162.00)	170.50	(28.00)	0.16
HDL-C (mg/dL)	47.00	(11.00)	46.00	(10.50)	51.00	(15.0)	0.22	32.00	(6.00)	34.00	(7.00)	32.00	(6.00)	0.13
sRAGE (pg/mL)	1919.48 ^d^	(1020.60)	1694.63	(895.60)	2645.47	(1809)	0.48	1989.21	(1311.00)	2222.37	(1240.60)	1924.14	(1020.20)	0.67

All values are expressed as median (M) and interquartile ranges (IQR). ^a^ TT vs. AA *p* = 0.046. ^b^ TA vs. AA *p* < 0.008. Significant differences according to multiple comparisons post-hoc analysis. ^c^ *n* = 41. ^d^ *n* = 43. WC = Waist circumference. BMI = Body mass index. SBP = Systolic blood pressure. DBP = Diastolic blood pressure. HDL-C = High-density lipoprotein cholesterol.

**Table 4:** Clinical and metabolic variables according to the genotypes of the −429 T/C polymorphism.

	Non-MS Group					MS Group				
	TT		TC		*p*	TT		TC		*p*
	*n* = 62		*n* = 17			*n* = 67		*n* = 12		
	Median	(IQR)	Median	(IQR)		Median	(IQR)	Median	(IQR)	
Age (years)	33.00	(8.00)	38.00	(10.00)	0.06	37.00	(10.00)	40.00	(10.00)	0.77
WC (cm)	84.75	(7.10)	86.30	(7.10)	0.25	101.00	(10.50)	101.50	(11.40)	0.35
BMI (kg/m^2^)	23.87	(2.80)	23.49	(2.60)	0.53	29.45	(5.00)	32.41	(5.60)	0.08
SBP (mg/dL)	109.00	(16.00)	110.00	(15.00)	0.75	129.00	(17.00)	120.50	(14.50)	0.19
DBP (mg/dL)	73.00	(11.00)	71.00	(6.00)	0.70	80.00	(12.00)	80.00	(9.50)	0.82
Glucose (mg/dL)	87.50	(16.00)	91.00	(14.00)	0.52	105.00	(14.00)	111.50	(14.00)	0.02 ^a^
Creatinine (mg/dL)	1.00	(0.20)	1.00	(0.20)	0.42	1.10	(0.20)	1.10	(0.20)	0.36
Triglycerides (mg/dL)	89.50	(50.00)	80.00	(40.00)	0.90	203.00	(115.00)	210.00 ^b^	(63.00)	0.84
HDL-C (mg/dL)	47.00	(11.00)	49.00	(12.00)	0.63	33.00	(6.00)	30.50	(10.00)	0.17
sRAGE (pg/mL)	1859.43 ^c^	(999.80)	1919.48	(767.10)	0.80	2013.04	(1278.00)	1805.00	(695.10)	0.35

Because only one sample with the CC genotype was observed in each group (healthy and metabolic syndrome subjects), the Mann-Whitney U test was used. All values are expressed as median (M) and interquartile ranges (IQR). ^a^ TT vs. TC *p* = 0.02. Significant difference according to multiple comparisons post-hoc analysis. ^b^ *n* = 11. ^c^ *n* = 60. WC = Waist circumference. BMI = Body mass index. SBP = Systolic blood pressure. DBP = Diastolic blood pressure. HDL-C = High-density lipoprotein cholesterol.

**Table 5:** Association of haplotypes with metabolic syndrome.

Haplotype	Frequency	Ratios MS, Non-MS	X^2^	*p*
TT	0.642	102.4:57.6, 103.0:57.0	0.004	0.95
AT	0.255	43.6:116.4, 38.0:122.0	0.51	0.48
TC	0.103	14.0:146.0, 19.0:141.0	0.845	0.36

## Data Availability

The data will be available on request from the corresponding author. The data are not publicly available due to it was not uploaded to a public database.
